# Single use and conventional bronchoscopes for Broncho alveolar lavage (BAL) in research: a comparative study (NCT 02515591)

**DOI:** 10.1186/s12890-017-0421-7

**Published:** 2017-05-05

**Authors:** Seher Raza Zaidi, Andrea M. Collins, Elena Mitsi, Jesús Reiné, Kayleigh Davies, Angela D Wright, Jessica Owugha, Richard Fitzgerald, Amitava Ganguli, Stephen B Gordon, Daniela Mulari Ferreira, Jamie Rylance

**Affiliations:** 1 Respiratory Infection Group, Liverpool School of Tropical Medicine, Pembroke Place, Liverpool, L3 5QA UK; 2 Respiratory Research Group, Royal Liverpool and Broadgreen University Hospital NHS Trust, Liverpool, L7 8XP UK; 3 Clinical Research Network, Northwest Coast, Liverpool, UK; 4 Malawi-Liverpool-Wellcome Trust Clinical Research Programme, Blantyre, Malawi

**Keywords:** Research Bronchoscopy, Bronchoalveolar Lavage, Single use bronchoscopes, Bronchoscopy, Bronchoalveolar lavage fluid, Lung diseases

## Abstract

**Background:**

Broncho alveolar lavage (BAL) is widely used for investigative research to study innate, cellular and humoral immune responses, and in early phase drug trials. Conventional (multiple use) flexible bronchoscopes have time and monetary costs associated with cleaning, and carries a small risk of cross infection. Single use bronchoscopes may provide an alternative, but have not been evaluated in this context.

**Methods:**

Healthy volunteers underwent bronchoscopy at a day-case clinical research unit using the Ambu® aScope^TM^ single-use flexible intubation bronchoscope. Broncho alveolar lavage was performed from a sub segmental bronchus within the right middle lobe; a total of 200 ml of warmed normal saline was instilled then aspirated using handheld suction. BAL volume yield, cell yield and viability were recorded.

**Results:**

Ten volunteers, (mean age 23 years, six male) participated. Bronchoscopies were carried out by one of two senior bronchoscopists, experienced in the technique of obtaining BAL for research purposes. The results were compared to 50 (mean age 23, 14 male) procedures performed using the conventional scope by the same two bronchoscopists. The total volume yield was significantly higher in the disposable group median 152 ml (IQR 141–166 ml) as compared to conventional 124 ml (110–135 ml), *p* = <0.01. The total cell yield and viability were similar in both groups, with no significant differences.

**Conclusions:**

With single use bronchoscopes, we achieved a larger BAL volume yield than conventional bronchoscopes, with comparable cell yield and viability. Better volume yields can potentially reduce post procedure side effects such as pleuritic chest pain and cough. The risk of cross infection can be eliminated, providing reassurance to researchers and participants. Reduced maintenance requirements can be cost effective. These could potentially be used for early phase drug development studies.

**Trial registration:**

This trial was registered prospectively in July 2015 with the National Clinical Trials register, with the following registration number assigned: NCT 02515591.

## Background

Flexible bronchoscopy is widely performed in adults and children for investigation of pulmonary pathology [1, 2]. Broncho alveolar lavage (BAL) sampling is used to study innate, cellular and humoral immune responses, determining the cell population profiles that can facilitate the diagnosis of various diffuse lung diseases [3–7]. It is used in early phase drug development studies and has a well-proven safety record in both research and in clinical applications. BAL is easily performed and well-tolerated with rare complications [8–10].

Typically, conventional flexible bronchoscopes are used but they are associated with significant costs related to initial purchase, ongoing maintenance, and sterilisation [11, 12]. Single use bronchoscopes offer an alternative [11] and are currently used in many UK NHS trusts for both emergency and elective airway intubations [13]. Single use scopes are more portable, and might also improve working efficiency [12]. Their efficacy for research studies has not yet been demonstrated, notably in research BAL the cell number and viability, and the returned volume of epithelial lining fluid is critical. Maximising the volume of BAL fluid returned has potential advantages to both researchers and participants: procedures that return less than 100 mL are more frequently associated with side effects such as cough, pleuritic chest pain and fever. Larger total instilled volumes of a minimum of 100 mL and a recommended standard 240 mL using standard 4 × 60 mL aliquots have therefore been recommended by the European Respiratory Society (ERS) to improve standardization when more efficient alveolar sampling and accurate quantitative measurements are required [14]. For cellular studies, function and viability are important, and may be maximised by the use of manual suction which minimises cellular shear forces [8]. Rapid processing by designated laboratory staff highly trained in handling of BALF (BAL fluid) samples is ideal. However, it should be noted that there is no strong relationship between the volumes returned and cell numbers obtained (unpublished data from our group).

This study presents a comparison of single use disposable bronchoscopes and conventional bronchoscopes with regards to BAL volumes, cell yields and viability using each method.

## Methods

The aim of the study was to compare the BAL volume yield, total cell yield and viability between samples obtained using single use and conventional bronchoscopes. Table 1 compares different features of the single use and flexible bronchoscopes.

**Table 1:** Features of Single-use and Multiple Use Bronchoscopes

		Multiple Use Bronchoscope	Single-use Bronchoscope
Optical Systems	Field of View	120°	85°
	Direction of View	Forward Viewing	Forward Viewing
	Depth of field	2–100 mm	8–19 mm
Insertion section	Distal end outer diameter	4.8 mm	5.4 mm
	Insertion tube outer diameter	4.9 mm	5.0 mm
	Working length	600 mm	600 mm
Instrument Channel		2.0 mm	2.0 mm
Risk of cross infection		Yes	No
Potential delay due to cleaning		Yes	No
Cost		Sterilisation Servicing Initial equipment cost	Repeated purchase cost
Portability		Depends on location of image processing unit	Can be hand held

### Recruitment

We enrolled healthy volunteers aged 18–55 years old, to undergo bronchoscopy using the Ambu® aScope^TM^ Regular 5.0/2.2 single-use flexible intubation bronchoscope. The study was carried out in the Clinical Research Unit (CRU) at the Royal Liverpool University Hospital (RLUH). The primary aim was to compare the BAL volume yield (mL), cell yield (total cell number) and proportion of viable cells (alveolar macrophages [AM] and lymphocytes), with recent data from procedures using conventional bronchoscopes. Conventional procedures were performed on 50 healthy volunteers recruited at the same site with identical inclusion and exclusion criteria. The demographics are described in Table 2.

**Table 2:** Demographics

	Single-use (*n* = 10)	Conventional (*n* = 50)
Age (yr) mean ± SD ^a^	23.4 ± 1.8	25.9 ± 4.2
Males (%)	6 (60%)	19 (37.2%)

^a^ Un-paired T-test

A physical examination including vital signs was performed. A detailed history of complications associated with other procedures or trauma was obtained, and risks for bleeding sought according to guidelines [7], specifically medications (e.g. clopidogrel, aspirin, Coumadin, heparin), and relevant medical conditions (e.g. uremia). Exclusion criteria were: a history of allergic reaction to benzodiazepines, or any anaesthetic agent; smoking history of > 10 pack years; any tobacco smoking in the preceding 3 months; pregnancy; abnormalities of screening blood tests (haemoglobin, white cell count, platelets, liver transaminases, bilirubin, renal and clotting profile).

### Bronchoscopy and Broncho alveolar lavage

Bronchoscopy was carried out as a day case according to previously published protocol [8]. Briefly, local anaesthesia was attained using topical lidocaine gel and spray, with further 4% lidocaine administered to the larynx and 2% lidocaine to the bronchial tree via the scope. Warmed 0.9% saline was instilled to the right middle lobe in sequential aliquots (60, 50 and 40 mL), with aspiration into a sterile syringe using gentle manual suction. BAL yields were recorded, and fluid transported immediately to the laboratory on melting ice [8]. We used continuous monitoring of heart rate, blood pressure and oxygen saturations during the procedure, with supplemental oxygen given by nasal cannula.

All procedures were carried out by one of two senior bronchoscopists, experienced in obtaining BAL for research purposes. Hospital procedures required that conventional bronchoscopy was performed in the surgical theatres, whereas flexible bronchoscopy was performed in the research ward: this was the only difference between the groups.

### Sample processing

BAL fluid (BALF) was filtered through double layered gauze to remove mucus plugs. Cells were pelleted by centrifugation (1500 rpm for 10 min at 4 °C), and washed with 50 mL cold RPMI medium (Gibco™ RPMI 1640 Medium) containing antibiotics (Penicillin, Neomycin and Streptomycin, Sigma-Aldrich, Sigma Chemical Co. St. Louis, MO, USA). The centrifugation step was repeated once, and the cell pellet was re-suspended in culture medium, with the addition of 10% FBS Gibco-Invitrogen (Life Technologies GmbH, Eggenstein, Germany). Cell suspensions were examined as five times diluted in trypan blue for counting and viability assessment using a haemocytometer.

### Statistical analysis

Primary outcome measures were compared with values from the preceding 50 conventional procedures using the Mann Whitney U Test. Statistical analyses were performed with Graph Pad Prism version 5.0, Graph Pad Software, La Jolla, CA, USA).

## Results

**Fig. 1:**
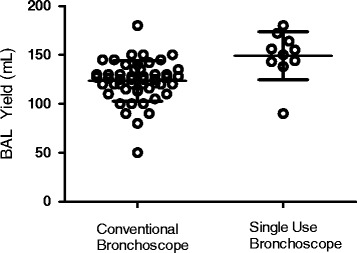
BAL Fluid Volume Yield (mL) from Conventional vs Single-use Bronchoscopes

**Fig. 2:**
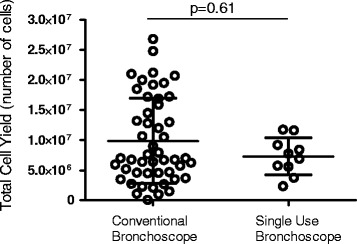
Total cell yield (no.) from Conventional vs Single-use Bronchoscopes

**Fig. 3:**
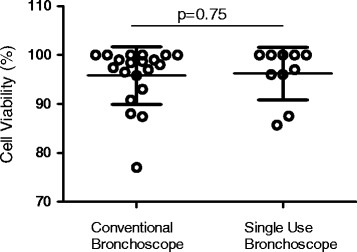
Cell Viability (%) from Conventional vs Single-use Bronchoscopes

Ten participants (6 male), mean age of 23.4 years (range 20–26 years) were enrolled. All participants were intubated nasally, and only one requested sedation (midazolam 3 mg used). The median BAL volume yield from the single-use bronchoscopes was 152 mL (IQR 141–166 mL) as compared to conventional 124 mL (110–135 mL), *p* < 0.01 (Fig. 1). The median total cell yield from single-use bronchoscopes was 7.33 × 10^6^ (5.13 × 10^6^–9.80 × 10^6^) compared with 7.0 × 10^6^ (4.53 × 10^6^–1.64 × 10^7^) for conventional procedures, *p* = 0.61 (Fig. 2). The median cell viability for samples from single use bronchoscopes was 98.5% (93.8–100) compared to 98.2% (93.7–100%), *p* = 0.75 (Fig. 3). The comparison for the demographics is described in Table 2.

## Discussion

Broncho alveolar lavage with single-use flexible bronchoscope achieved greater BAL volume yields than with conventional bronchoscopes. There was no significant difference between the cell yield and viability between the methods.

Single-use bronchoscopes have been evaluated in the critical care setting with favourable evaluation for bronchoscopy, tracheostomy, intubation and suction [11]. Our group has experience of over 1500 research BALs, and the procedures in this study were performed by senior bronchoscopists with extensive experience of BAL for research purposes; allowing good comparison with the use of conventional bronchoscopes. BAL volume yields were similar in male and female participants in our study, as seen in other studies [15].

The greater BAL volume return achieved with single-use bronchoscopes could lead to reduced risk of post-procedural side effects such as cough, pleuritic chest pain and fever, which may improve tolerability and participant comfort. However, we have not systematically collected these data.

Single use flexible bronchoscopes have the potential for use in pharmaceutical preclinical and clinical studies for medicine development.

## Conclusion

Single-use flexible bronchoscopes can be used to obtain BAL for research purposes to study immune responses and in early phase drug development studies.

## Abbreviations

BAL: Broncho alveolar Lavage
RML: Right Middle Lobe bronchus
